# Activation of the integrated stress response (ISR) pathways in response to Ref-1 inhibition in human pancreatic cancer and its tumor microenvironment

**DOI:** 10.3389/fmed.2023.1146115

**Published:** 2023-04-27

**Authors:** Mahmut Mijit, Megan Boner, Ricardo A. Cordova, Silpa Gampala, Eyram Kpenu, Angela J. Klunk, Chi Zhang, MarK R. Kelley, Kirk A. Staschke, Melissa L. Fishel

**Affiliations:** ^1^Department of Pediatrics and Herman B Wells Center for Pediatric Research, Indianapolis, IN, United States; ^2^Indiana University Simon Comprehensive Cancer Center, Indiana University School of Medicine, Indianapolis, IN, United States; ^3^Department of Biochemistry and Molecular Biology, Indiana University School of Medicine, Indianapolis, IN, United States; ^4^Department of Medical and Molecular Genetics, Indiana University School of Medicine, Indianapolis, IN, United States; ^5^Department of BioHealth Informatics, Indiana University School of Medicine, Indianapolis, IN, United States; ^6^Department of Pharmacology and Toxicology, Indiana University School of Medicine, Indianapolis, IN, United States

**Keywords:** pancreatic ductal adenocarcinoma (PDAC), integrated stress response (ISR), PERK, eIF2, Ref-1, redox signaling, hypoxia, tumor microenvironment

## Abstract

Pancreatic cancer or pancreatic ductal adenocarcinoma (PDAC) is characterized by a profound inflammatory tumor microenvironment (TME) with high heterogeneity, metastatic propensity, and extreme hypoxia. The integrated stress response (ISR) pathway features a family of protein kinases that phosphorylate eukaryotic initiation factor 2 (eIF2) and regulate translation in response to diverse stress conditions, including hypoxia. We previously demonstrated that eIF2 signaling pathways were profoundly affected in response to Redox factor-1 (Ref-1) knockdown in human PDAC cells. Ref-1 is a dual function enzyme with activities of DNA repair and redox signaling, responds to cellular stress, and regulates survival pathways. The redox function of Ref-1 directly regulates multiple transcription factors including HIF-1α, STAT3, and NF-κB, which are highly active in the PDAC TME. However, the mechanistic details of the crosstalk between Ref-1 redox signaling and activation of ISR pathways are unclear. Following Ref-1 knockdown, induction of ISR was observed under normoxic conditions, while hypoxic conditions were sufficient to activate ISR irrespective of Ref-1 levels. Inhibition of Ref-1 redox activity increased expression of p-eIF2 and ATF4 transcriptional activity in a concentration-dependent manner in multiple human PDAC cell lines, and the effect on eIF2 phosphorylation was PERK-dependent. Treatment with PERK inhibitor, AMG-44 at high concentrations resulted in activation of the alternative ISR kinase, GCN2 and induced levels of p-eIF2 and ATF4 in both tumor cells and cancer-associated fibroblasts (CAFs). Combination treatment with inhibitors of Ref-1 and PERK enhanced cell killing effects in both human pancreatic cancer lines and CAFs in 3D co-culture, but only at high doses of PERK inhibitors. This effect was completely abrogated when Ref-1 inhibitors were used in combination with GCN2 inhibitor, GCN2iB. We demonstrate that targeting of Ref-1 redox signaling activates the ISR in multiple PDAC lines and that this activation of ISR is critical for inhibition of the growth of co-culture spheroids. Combination effects were only observed in physiologically relevant 3D co-cultures, suggesting that the model system utilized can greatly affect the outcome of these targeted agents. Inhibition of Ref-1 signaling induces cell death through ISR signaling pathways, and combination of Ref-1 redox signaling blockade with ISR activation could be a novel therapeutic strategy for PDAC treatment.

## 1. Introduction

Pancreatic ductal adenocarcinoma (PDAC) is one of the deadliest cancers, with a 5-years overall survival of approximately 11% (1). Currently, PDAC has highest mortality-to-incidence ratio amongst all malignancies, and it is projected to be the second leading cause of cancer-related deaths in the United States by 2030 (2). The vast majority of PDAC patients present with late-stage metastatic disease, inherent drug resistance, and high rate of recurrence (3).

PDAC tumor microenvironment (TME) is characterized by a dense stroma, consisting of cancer-associated fibroblasts (CAFs) and immunosuppressive cell populations, which remains a barrier for developing effective treatments (4). CAFs are the most abundant component of PDAC TME. Through multiple pathways, activated CAFs can promote tumor growth, angiogenesis, invasion, and metastasis, along with extracellular matrix (ECM) remodeling and even chemoresistance (3). Chemotherapy regimens, including gemcitabine used in combination with Abraxane or FOLFIRINOX, are frequently used as standard of care for PDAC and yet are ultimately rarely effective (5). Hence, there is an urgent need to identify novel therapeutic approaches to improve PDAC treatment and understand how the TME impacts upon treatment response (6).

We have been studying a protein highly expressed in both the tumor and the TME that has been implicated in the cellular response to stress such as hypoxia, DNA damage, inflammation, and metabolism: Apurinic/apyrimidinic endonuclease/reduction-oxidation factor 1 (APE1/Ref-1 or Ref-1). The endonuclease activity of APE1 is critical for repairing damaged DNA and/or RNA to maintain genome stability (7). Ref-1 redox signaling has been implicated in cancer as well as multiple other human diseases (1). The redox activity of Ref-1 regulates numerous transcription factors (TFs), including signal transducer and activator of transcription factor 3 (STAT3), activator protein 1 (AP-1), hypoxia-inducible factor (HIF-1α), nuclear factor κB (NF-κB), p53, and others, all known to be involved in cell growth, inflammation, and tumor metastasis (8). Targeting Ref-1 redox activity with small molecule inhibitors, such as APX3330 or APX2009 significantly blocked the activation of oncogenic TFs, and subsequently inhibited PDAC cell growth both *in vivo* and *in vitro* (1, 6, 9). However, adaptive mechanisms of resistance eventually emerge with targeted therapy, therefore elucidation of tumor response following Ref-1 inhibition is critical to identifying novel combinations and combatting this resistance (10).

**FIGURE 1:**
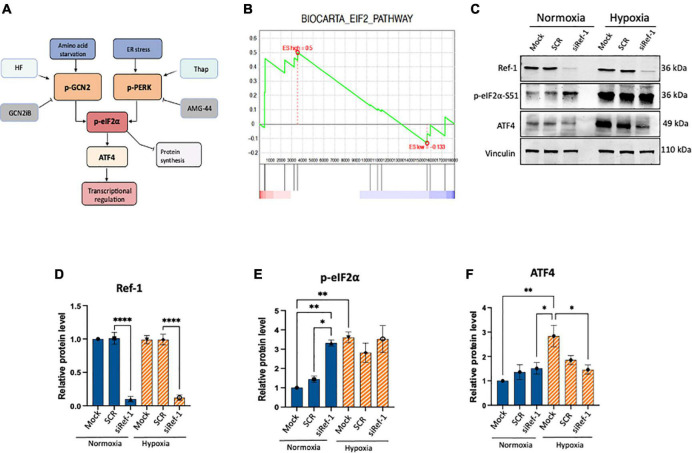
Activation of eIF2α was observed under conditions of Ref-1 KD or hypoxia in human pancreatic ductal adenocarcinoma (PDAC) line, Pa03C. **(A)** Schematic of integrated stress response (ISR) pathway kinases, general control non-derepressible 2 (GCN2) and protein kinase R-like endoplasmic reticulum kinase (PERK) and subsequent downstream proteins. Halofuginone (HF) activates the ISR *via* GCN2 while GCN2iB is an inhibitor of signaling through GCN2. Thapsigargin (Thap) activates the ISR through PERK and AMG-44 is an inhibitor of p-PERK. **(B)** Gene set enrichment analysis (GSEA) plot of single cell RNA seq analysis demonstrating eIF2 signaling pathway was enriched in Pa03C cells transfected with Ref-1 siRNA under normoxia (*p* < 0.01). **(C)** Expression levels of ISR biomarkers after Ref-1 KD under normoxia and hypoxia (1% O_2_, 24 h). **(D–F)** Quantification of western blots, *n* = 3, One-way analysis of variance (ANOVA), *p < 0.05, **p < 0.01, ****p < 0.0001 vs. Mock. SCR is referring to scrambled control; KD, knockdown.

Using single cell RNA-sequencing (scRNA-seq) following transfection of pancreatic cancer cells with Ref-1 siRNA, the top pathway that was significantly altered was eIF2 pathways (11). These data led to the study presented here where the role of the integrated stress response (ISR) following Ref-1 inhibition was investigated both in tumors and CAFs. The ISR is critical for cell adaptation and survival to environmental stresses (12). ISR pathway is initiated upon phosphorylation of the alpha subunit of eukaryotic initiation factor 2 (eIF2α) in response to diverse stress conditions. There are four eIF2 kinases that phosphorylate eIF2α: (1) general control non-derepressible 2 (GCN2), which is upregulated by amino acid starvation; (2) protein kinase R (PKR), which is activated by viral infections; (3) PKR-like endoplasmic reticulum (ER) kinase (PERK), which is upregulated by ER stress; (4) heme-regulated eIF2α kinase (HRI), which is induced upon oxidative stress or mitochondrial dysfunction (Figure 1A). Phosphorylated eIF2α (p-eIF2α) markedly attenuates translation initiation and overall protein synthesis in the cells. In addition, p-eIF2α facilitates the preferential translation of certain mRNAs, most notably ATF4, a transcriptional regulator of genes involved in amino acid metabolism and proteostasis control, oxidative stress defenses, along with feedback control of the ISR (13, 14). Therefore, gene reprograming by p-eIF2α functions in cell adaptation in response to mild stress and in promoting apoptosis or cell death in response to acute stress. In these studies, we utilized two inhibitors of ISR kinases, GCN2iB and AMG-44, both type I 1/2 ATP-competitive inhibitors of GCN2 and PERK, respectively (14–16).

In addition to PDAC TME heterogeneity, PDAC is also known for its ability to survive in a hypoxic environment. Multiple studies have investigated the adaptation of PDAC cells to the hypoxic microenvironment through HIF-1α signaling and the ISR (17–19). scRNA-seq implicated eIF2 signaling pathways as important following Ref-1 knockdown, however the tumor’s response to Ref-1 inhibition and subsequent activation of ISR pathways has not been characterized (11). In the present study, we evaluated the effects of Ref-1 redox blockade on ISR signaling in human PDAC cells and CAFs and demonstrated profound activation of ISR pathways in multiple lines. Activation of ISR signaling following drug treatment or cellular stress can be an adaptive response toward homeostasis that protects the cell from death or in contrast, sustained activation can lead to activation of apoptosis (20). In order to delineate which role the ISR was playing following Ref-1 inhibition, we used various activators and inhibitors of the pathway. Following knockdown of each of the ISR kinases, the ability to induce phosphorylation of eIF2 after Ref-1 inhibition was most effectively blocked when PERK expression was decreased. Finally, the cell killing effects of Ref-1 redox inhibitors in combination with PERK or GCN2 activators and inhibitors in 3D co-culture systems were evaluated. We demonstrated that tumor killing effects were greater with the combination of Ref-1 blockade and ISR activation suggesting that the prolonged activation of ISR is leading to cell death. CAFs appeared more responsive to combination treatment compared to tumor cells, again implicating Ref-1 redox signaling in a pro-survival role. Our data indicates the activation of ISR as being part of the mechanism of cell death in the tumor and TME in response to Ref-1 inhibition and the importance of using 3D co-culture systems in pancreatic cancer studies to study cellular stress response.

## 2. Materials and methods

### 2.1. Cell culture

Pa03C, 10.05, Pa02C, and CAF19 cells were obtained from Dr. Anirban Maitra at The Johns Hopkins University (21). HEK-293 (Cat. #CRL-3216, RRID:CVCL_0063) cells were purchased from the American Tissue Type Collection (ATCC, Manassas, VA, USA). The cells were cultured with DMEM (Invitrogen; Carlsbad, CA, USA) supplemented with 10% FBS (Hyclone; Logan, UT, USA) under 37°C in 5% CO_2_. The cells were placed in a hypoxia workstation (Ruskinn Invivo2 200) to generate hypoxic environment (1% O_2_, 24 h) as previously described (6, 22). The cell lines were authenticated by short tandom repeat (STR) analysis and confirmed to be mycoplasma negative.

### 2.2. Inhibitors treatment

Small molecule Ref-1 inhibitors APX3330, APX2009, and APX2014 (Apexian Pharmaceuticals) were prepared in DMSO as previously described (1). RN7-58 was used as a negative control and is structurally similar, but does not inhibit Ref-1 redox signaling activity (6). GCN2 inhibitor (GCN2iB) or activator Halofugione (HF) or PERK inhibitor (AMG-44) were used in combination with Ref-1 inhibitors. All inhibitors are dissolved in 100% DMSO. Specific details of inhibitors were provided in Supplementary Table 1.

### 2.3. Cell viability and cytotoxicity

For monolayer cell culture, cell proliferation and viability were measured with Alamar Blue Cell Viability assay (Invitrogen, Eugene, OR, USA) as previously described (1, 9). Cancer cell lines were seeded at 2,000 cells/well in 96-well tissue culture plates and cell viability was measured 48 h after treatment with various inhibitors. The cellular responses were normalized to a non-treated (media only or vehicle) control. At least three replicates were performed.

### 2.4. Tumor spheroid 3−dimensional (3D) assay

Pa03C and Pa02C cells express TdTomato and CAF19 express EGFP to enable us to track their growth over time. Cells were grown in co−culture as 3D tumor spheroids as previously described (6). The total intensity of the red or green signal from the spheroids over time was quantitated as described in our previous studies (6, 22). Briefly, PDAC and CAF (Cancer Associated Fibroblasts) cells (500:2,000 cell/well, 1:4 ratio) were seeded in ultralow adherence 96−well plates (Corning Inc.) in media containing 5% FBS and 3% reduced growth factor Matrigel (Corning Inc.). Spheroids were fed or treated on days 4, 8, and 11 with fluorescent intensity measured on days 4, 8, 11, and 14 following plating using the Thermo ArrayScan (Thermo Fisher Scientific) as previously described. Fold change was calculated to assess the effect of drug treatment on spheroid growth and was calculated compared to media control total intensity on Day 14 (23).

### 2.5. Western blotting analysis

Cells were lysed in 1% SDS extraction buffer supplemented with protease inhibitors (Santa Cruz Biotechnology, TX, USA). Briefly, cell extract was heated at 95°C for 5 mins, then sonicated (4 pulses, 4 cycles) to shear the DNA in the samples as previously described (1). Denatured samples (20–40 μg) were subjected to SDS-PAGE and proteins were transferred onto nitrocellulose membranes by electrophoretic transfer. Non-specific binding sites were blocked at room temperature for 1 h with 5% (w/v) Blotting-Grade milk (Bio-Rad Laboratories, CA, USA) in Tris–buffer saline (Boston Bio Products, MA, USA) containing 0.05% (v/v) Tween-20 (Thermo Fisher, MA, USA) (TBS-T). Membranes were incubated overnight with the primary antibodies, and then with the peroxidase-conjugated secondary antibody for 1 h (Supplementary Table 2). Signal was then captured by using Bio-Rad ChemiDoc imager, and band intensities were analyzed by densitometry on Image Lab (Bio-Rad Laboratories, CA, USA) or ImageJ software.

### 2.6. siRNA transfections

Cells were transfected by lipofectamine RNAiMax (Invitrogen, CA, USA) with targeted siRNAs or universal scrambled control (SCR) siRNAs (Supplementary Table 3). After transfection, the cells then treated with Ref-1 inhibitors (APX2009) for 6 h. Knock-down (KD) efficiency of target proteins was verified by Western blot or qPCR.

### 2.7. RNA isolation, reverse transcription, and real-time quantitative PCR (qRT-PCR)

Cells were collected and processed for RNA extraction according to the manufacturer’s protocol (Qiagen, Hilden, Germany, USA). The RNA concentrations were determined using a NanoDrop (Thermo Fisher, MA, USA). Subsequently, 1 μg of RNA/25-μl reaction mix was reverse-transcribed to cDNA using (Applied Biosystems, Warrington, UK). qRT-PCR was performed in 96-well plates, with a final volume of 20 μL/well using the SYBR Green PCR kit (Applied Biosystems, Foster City, CA, USA) on the CFX96 real-time PCR detection system (BioRad, Hercules, CA, USA). Primers for indicated genes are commercially available (OriGene, Technologies, MD, USA) and primers sequence are shown in Supplementary data (Supplementary Table 4). qRT-PCR cycling conditions were 1 min at 95°C, 10 min at 95°C, 15 s at 95°C and 1 min at 60°C for 40 cycles. Relative changes in mRNA expression levels were assessed by the 2^–ΔΔ*CT*^ method, and changes in mRNA expression of the target gene were normalized to that of β-actin gene (1, 24).

### 2.8. ATF4 luciferase activity

The ATF4 reporter cell line was constructed by transducing HEK293A cells with a lentivirus encoding an ATF4 luciferase reporter gene consisting of six copies of an ATF4-C/EBPβ binding element (5’-GCGGGGATGATGCAATGTT-3’) upstream of a minimal promoter followed by the firefly luciferase coding sequence. The reporter cells were maintained in DMEM supplemented with 10% FBS and 1 ug/ml puromycin. To measure ATF4 transcriptional activity, reporter cells were seeded into 96-well plates at 15,000 cells per well and allowed to attach overnight. Cell treatments were at 37°C for 6 h, and luciferase activity was measured using Bio-Glo Luciferase Assay Reagent (Promega Cat. #G7940) using a Synergy H1 Multi-Mode Microplate reader (BioTek, Winooski, VT, USA).

### 2.9. Bioinformatic analysis

We utilized our in-house generated scRNA-seq data of Ref-1 knockdown (si-Ref-1) and scrambled control (SCR) Pa03C cells under normoxia and hypoxia conditions (11). Differential gene expression analysis was conducted by using Left Truncated Mixture Gaussian models with FDR < 0.05 as the significant cutoff (25). Pathway enrichment analysis was conducted by using Gene Set Enrichment Analysis (GSEA) against MsigDB v6 canonical pathways (26). GSEA plot and enrichment scores of the expression variation in siRef-1 vs. SCR were visualized for selected pathways.

### 2.10. Statistical analysis

All experiments were performed at least three independent times and the data obtained were expressed as “Mean and Standard Error.” Significance was calculated using one-way ANOVA where applicable using Graph Pad Prism Version 9 as previously described (1, 6). Statistical significance was considered when the *p*-value < 0.05. For synergy calculations, zero interaction potency models (ZIP) was used (27) and Calcusyn was used. The ZIP model takes into account the change in potency for single agents compared to their combination. The web-based platform, SynergyFinder 3.0 was used to calculate the values here^1^ and generate the synergy maps. According to the model, a score of −10 or lower indicates antagonism, −10 to 10 indicates additivity, and a score of 10 or greater is synergy. These guidelines were followed for the combination drug studies presented here.

## 3. Results

### 3.1. Activation of eIF2α was observed under conditions of Ref-1 KD or hypoxia

Tumor cells can adapt and survive under harsh conditions and a variety of cellular stresses and the ISR pathway is important in this response (Figure 1A). However, crosstalk between Ref-1 redox signaling and ISR pathways has not been established. Previous work from our lab investigated the impact on gene expression using scRNA-seq analysis under conditions of hypoxia and Ref-1 knockdown (KD), and pathway analysis demonstrated eIF2 signaling to be the top dysregulated pathway under normoxia (11). Gene Set Enrichment Analysis (GSEA) demonstrates that knockdown of Ref-1 in Pa03C PDAC cells significantly upregulates genes corresponding to the eIF2 signaling pathway in normoxia (*p* < 0.01) (Figure 1B).

To further explore and validate these effects, we assessed the expression levels of ISR markers following Ref-1 KD both under normoxia and hypoxia (Figures 1C–F, Supplementary Figure 1). Protein expression data demonstrated that Ref-1 KD resulted in significantly increased phosphorylation of eIF2α under normoxia as well as significant induction of ISR in hypoxia (Mock normoxia vs. Mock hypoxia, *p* < 0.05, Figures 1C–F). We also validated the transcriptomic data showing that Ref-1 KD activates the ISR under normoxia (normoxia: SCR vs. siRef-1, *p* < 0.05), while hypoxic conditions were sufficient to activate eIF2 signaling irrespective of Ref-1 levels (hypoxia: SCR vs. siRef-1, *p* > 0.05), which was in line with the data that we observed from our single cell RNA studies (6, 11).

### 3.2. Inhibition of Ref-1 redox signaling results in activation of integrated stress response in pancreatic cancer cells

To examine if ISR is an active signaling pathway in human pancreatic cancer cells, we treated pancreatic cancer line, Pa03C with two well-characterized ISR activators, Halofuginone (HF) and Thapsigargin (Thap), which are specific activators of GCN2 and PERK, respectively (Figure 1A; 28–30). We demonstrate the clear time-dependent phosphorylation of p-GCN2 and p-PERK following HF and Thap within 0.5–6 h treatment. Accordingly, expression of p-eIF2α and its downstream target ATF4 were also increased in a time-dependent manner (Supplementary Figure 2).

**FIGURE 2:**
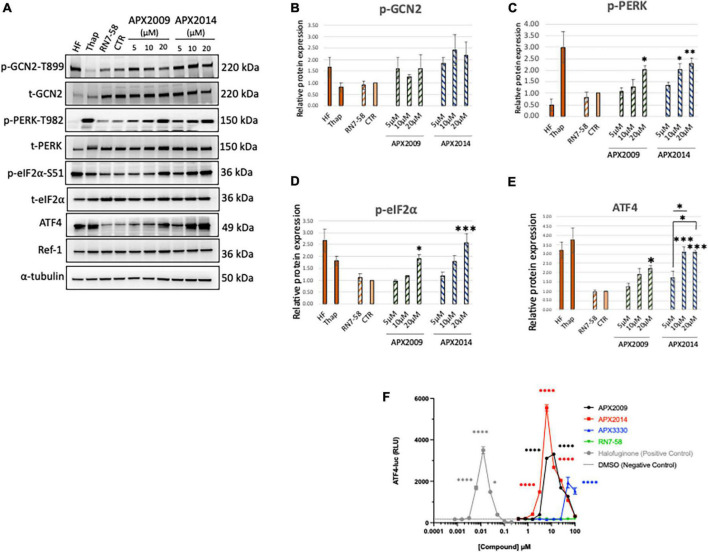
Inhibition of Ref-1 redox signaling results in activation of integrated stress response in human pancreatic cancer cells. **(A)** Human pancreatic cancer cells (Pa03C) were treated with increasing concentrations of Ref-1 redox inhibitors, APX2009 and APX2014 for 6 h. RN7-58 (20 μM, 6 h) is a negative control analog, which does not inhibit Ref-1 redox activity. DMSO was used as vehicle control (CTR) for the experiments. Cells treated with HF (1 nM) and Thap (100 μM) for 6 h were used as positive control for activation of integrated stress response (ISR) signaling pathways. Expression for various ISR proteins was evaluated following treatment in Pa03C cells and a representative image is shown. α-tubulin was used as loading control. **(B–E)** Quantification of expression levels of ISR markers. One-way analysis of variance (ANOVA) was used for statistical analysis, asterisk «*» is in comparison to CTR (DMSO): *p < 0.05, **p < 0.01, ***p < 0.001. At least three independent experiments were performed (*N* = 3–4). **(F)** HEK293 cells were treated with Ref-1 inhibitors for 6 h and ATF4 luciferase activity was assessed, *N* = 2. Compounds were tested at the indicated concentrations, and error bars indicate standard error of the mean (SEM). Statistical significance was determined using an ordinary one-way analysis of variance (ANOVA) with Dunnett’s multiple comparisons test, with a single pooled variance; *p ≤ 0.05, ****p ≤ 0.0001.

The Ref-1 KD transcriptomic and molecular data did demonstrate activation of eIF2α, however this approach reduces levels of the entire Ref-1 protein. This results in a reduction in both of its activities: DNA repair as well as redox signaling. We have developed redox signaling inhibitors that do not impact the DNA repair activity of Ref-1 which can be used to evaluate the pancreatic cancer cells’ response which are specific to a block in redox signaling (1, 6, 9). Following treatment with increasing concentrations of Ref-1 redox inhibitors, APX2009 and APX2014 along with the inactive analog RN7-58, relative expression of ISR specific kinases and downstream effectors were evaluated in Pa03C cells (Figure 2A).

We demonstrated that both Ref-1 inhibitors APX2009 and APX2014 induced clear dose-dependent activation of ISR kinase, p-PERK, but to a lesser extent p-GCN2 in Pa03C cells (Figures 2B, C < 0.05, DMSO vs. 20 μM APX2009/APX2014). The induction was stronger with APX2014, which is a newer Ref-1 analog and potentially more potent than APX2009 (31). Interestingly, inactive analog, RN7-58 failed to induce similar effects on the ISR pathway in Pa03C cells (*P* > 0.05, DMSO vs. RN7-58), suggesting that the activation of ISR kinases is specific to the inhibition of Ref-1 redox signaling and not due to non-specific effects of drug treatment. Positive control compounds, HF and Thap are included in the analysis as a reference for pathway activation (Figure 2). In addition, we also demonstrated that both APX2009 and APX2014 resulted in dose-dependent activation of p-eIF2α and its downstream target, ATF4 upon similar experimental conditions (Figures 2D, E). The induction was significantly higher when cells were treated with higher concentrations of Ref-1 inhibitors (*p* < 0.05, DMSO vs. 20 μM APX2009/APX2014, Figures 2D, E). Again, we did not observe any effect on p-eIF2α or ATF4 levels when cells were treated with RN7-58. We also showed that the expression of Ref-1 protein levels remains constant under these conditions (*p* > 0.05, DMSO vs. APX2009/APX2014). We evaluated the effects of Ref-1 inhibitors in additional human PDAC cell lines, Pa02C and Panc10.05 and confirmed the dose-dependent activation of ISR in multiple human PDAC lines following inhibition of Ref-1 (Supplementary Figure 3).

Finally, we examined the effect of Ref-1 redox inhibitors (APX3330 (parent compound), APX2009, APX2014) along with RN7-58 on ATF4 luciferase activity in HEK293 stable line (Figure 2F). We demonstrated that there is significant induction in ATF4 transcriptional activity, the effects were 1∼3-fold greater with APX2014 compared to APX2009 and APX3330. Similar to the results in Pa03C cells, there was no ATF4 activation with RN7-58 treatment (Figure 2F). Taken together, these results clearly point out that inhibition of Ref-1 redox signaling results in activation of the ISR in multiple cell types.

### 3.3. Multiple ISR kinases are involved in Ref-1/eIF2/ATF4 axis, preferentially through PERK

**FIGURE 3:**
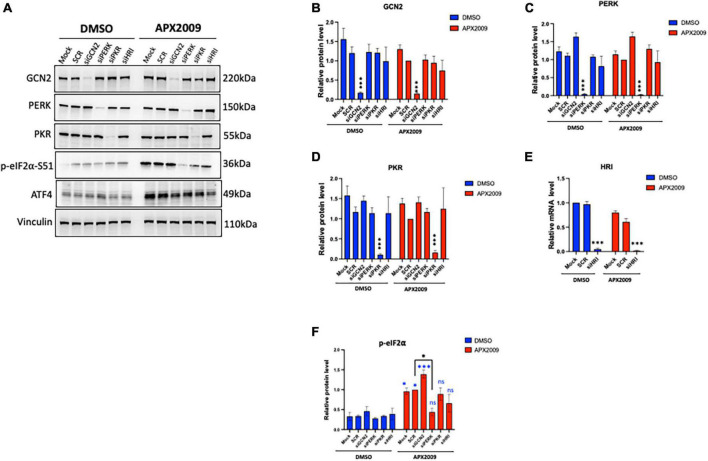
Multiple integrated stress response (ISR) kinases involved in Ref-1/eIF2α/ATF4 axis, preferentially through protein kinase R-like endoplasmic reticulum kinase (PERK). ISR kinases (GCN2, PERK, PKR, and HRI) were selectively knocked down in human pancreatic ductal adenocarcinoma (PDAC) cells (Pa03C) for 48 h, then the cells were treated with APX2009 (20 μM) for 6 h. **(A)** Representative Western blot image of ISR kinase protein expression levels after indicated knockdown. **(B–F)** Quantification of expression levels of ISR proteins or mRNA levels following APX2009 treatment. HRI knockdown efficiency was confirmed by qPCR panel **(E)**. One-way analysis of variance (ANOVA), *p < 0.05, ****p < 0.0001. *N* = 3–4. “*” in blue color comparing to DMSO. “ns” is referring to statistically non-significant. SCR is referring to scrambled control; KD, knockdown.

To determine which kinase upstream of eIF2α is driving the effects of ISR activation following Ref-1 inhibition, GCN2, PERK, PKR as well as HRI were knocked down in human pancreatic cancer cells, Pa03C using siRNA and subsequently treated with APX2009 for 6 h. Reduced expression of each eIF2 kinase was confirmed by Western blotting or qPCR (Figures 3A–E). We again confirmed that there is significant induction of p-eIF2α following APX2009 treatment (*p* < 0.05, Mock/SCR DMSO vs. APX2009, Figure 3F), as in Figure 2D. Interestingly, such effects of APX2009 were significantly decreased when PERK expression was knocked down (APX2009: SCR vs. PERK KD, *p* < 0.05), with p-eIF2α expression similar to control (DMSO: PERK KD vs. APX2009: PERK KD, *p* > 0.05). Similar results were found with PKR and HRI KD as APX2009 failed to strongly induce p-eIF2α (DMSO vs. APX2009, *p* > 0.05), although the effects were greater with PERK KD. Increased p-eIF2α following Ref-1 inhibitor treatment was not affected by GCN2 KD. Taken together, we demonstrated that blockade of Ref-1 redox signaling results in ISR activation, which is due to multiple ISR kinases but preferentially through PERK and not GCN2.

### 3.4. Inhibition of PERK with AMG-44 resulted in GCN2-dependent activation of the ISR on multiple human PDAC cells

**FIGURE 4:**
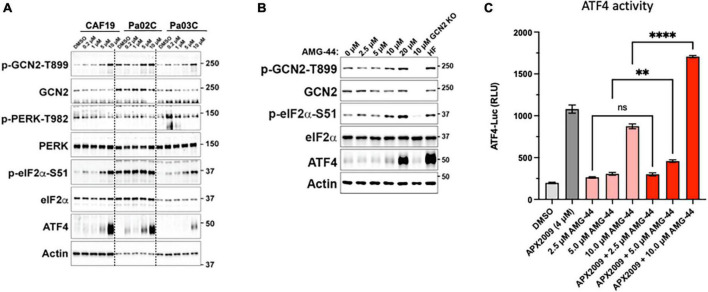
Differential response on activation of the integrated stress response (ISR) to protein kinase R-like endoplasmic reticulum kinase (PERK) inhibitor, AMG-44 based on concentration. Cancer-associated fibroblasts (CAF19) and pancreatic ductal adenocarcinoma (PDAC) cells, Pa02C and Pa03C **(A)** or 293A-ATF4-luc reporter cells **(B)** were treated with increasing concentrations of AMG-44 for 6 h. Expression levels of ISR signaling proteins as indicated were assessed by immunoblot, and a representative image is shown for each. Actin was used as loading control. Molecular weight markers are indicated in kilodaltons for the panels. **(C)** HEK293A-ATF4-luc reporter cells were treated with 5 to 20 μM AMG-44 or vehicle control (DMSO) for 6 h and luciferase activity was measured. Statistical significance was determined using an ordinary one-way analysis of variance (ANOVA) with Dunnett’s multiple comparisons test with a single pooled variance. Error bars indicate SE (*N* = 3); **p ≤ 0.001, ****p ≤ 0.0001.

**FIGURE 5:**
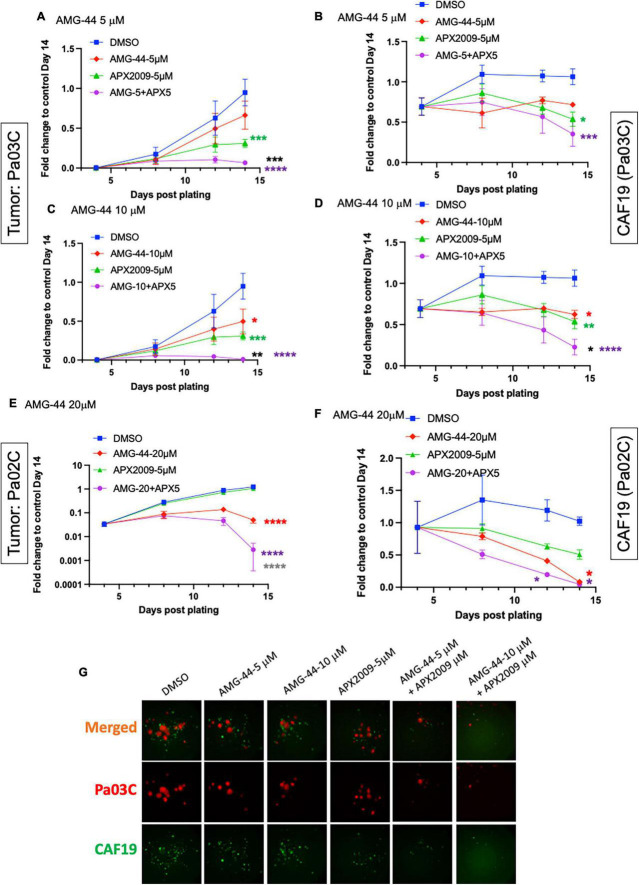
Combination treatment with Ref-1 and protein kinase R-like endoplasmic reticulum kinase (PERK) inhibitors results in enhancement of spheroid growth inhibition, but only at doses that demonstrated activation of the integrated stress response (ISR) in physiological relevant 3D culture system. Co-cultures of Pa03C + CAF19 **(A–D)** or Pa02C + CAF19 **(E,F)** are plated, scanned, and treated on Days 4, 8, and 11. Final scan is on Day 14. Quantitation of the fluorescent intensity of tumor cells expressing TdTomato, and cancer-associated fibroblasts (CAFs) expressing EGFP is shown over time. Normalization of total intensity is done by comparing treated well-intensity to Media control wells on Day 14. **(G)** Representative images of 3D co-culture assays with Pa03C (red) + CAF19 (green) cells following treatment with AMG-44 (5 and 10 μM) in combination with APX2009 (5 μM). Error bars indicate SE (*N* = 3); Two way analysis of variance (ANOVA) used to generate *p ≤ 0.05, **p ≤ 0.01, ***p ≤ 0.001, ****p ≤ 0.0001, colored symbols is in comparison to DMSO, black symbols are in comparison to AMG-44 treatment alone, and dark gray symbols are in comparison to APX2009 treatment alone.

To investigate the role of PERK in cellular response to Ref-1 inhibition in both the tumor and CAFs as a representation of cells from the PDAC microenvironment, PERK inhibitor AMG-44 was utilized alone and in combination with Ref-1 inhibitors. We hypothesized that blocking the ability of PERK to phosphorylate eIF2α using AMG-44 might lead to an increase in Ref-1-mediated cytotoxicity. As anticipated, AMG-44 is a potent inhibitor of PERK and the ISR at lower concentrations (Supplementary Figure 4). Interestingly, treatment of CAF19, the PDAC cell lines Pa02C and Pa03C, or the ATF4 reporter cells with AMG-44 induced rather than inhibited the ISR at higher concentrations (Figures 4A–C). The increase in ATF4 protein and transcriptional activity was dependent on GCN2 (Figure 4B and Supplementary Figure 5). We next utilized our co-culture 3D assay to determine the effects of PERK and Ref-1 inhibition on 3D spheroid growth. Based on the ATF4 activity data in Figure 4, concentrations of AMG-44 that blocked PERK activity as well as concentrations resulting in GCN2 activation were used in the 3D co-culture assay to evaluate spheroid growth. At low concentrations of AMG-44 where PERK activity was blocked, there was no benefit of combination treatment in the 3D spheroids consisting of two PDAC lines, Pa03C or Pa02C in co-culture with CAF19 cells (Supplementary Figure 6). However, in contrast, at concentrations of AMG-44 that demonstrated activation of ATF4 activity through GCN2, spheroid growth was significantly blocked when both compounds were present (Figure 5, single agents red and green curves and combination in purple). In Pa03C cells, there is a dose-dependent increase in tumor (Figures 5A, C) and CAF (Figures 5B, D) cell killing with combination therapy of AMG-44 + APX2009 (Figure 5G). Using Bliss or HSA analysis through Synergy Finder 3.0, the effects were synergistic in tumor cells (> 10) as well as CAFs (> 10). Similar results were observed for Pa02C cells, although these cells were more resistant to both Ref-1 and PERK inhibitor (Figures 5E, F) and the results of Bliss or HSA analysis indicated additivity (with a range from −5.6 to 2.8) for both tumors and CAFs, respectively. Taken together, these results suggest that activation of ISR through GCN2 contributes to the inhibitory activity of Ref-1 inhibition in our pancreatic cancer co-culture models.

### 3.5. Cell killing following inhibition of Ref-1 redox signaling is enhanced when ISR is activated through GCN2

**FIGURE 6:**
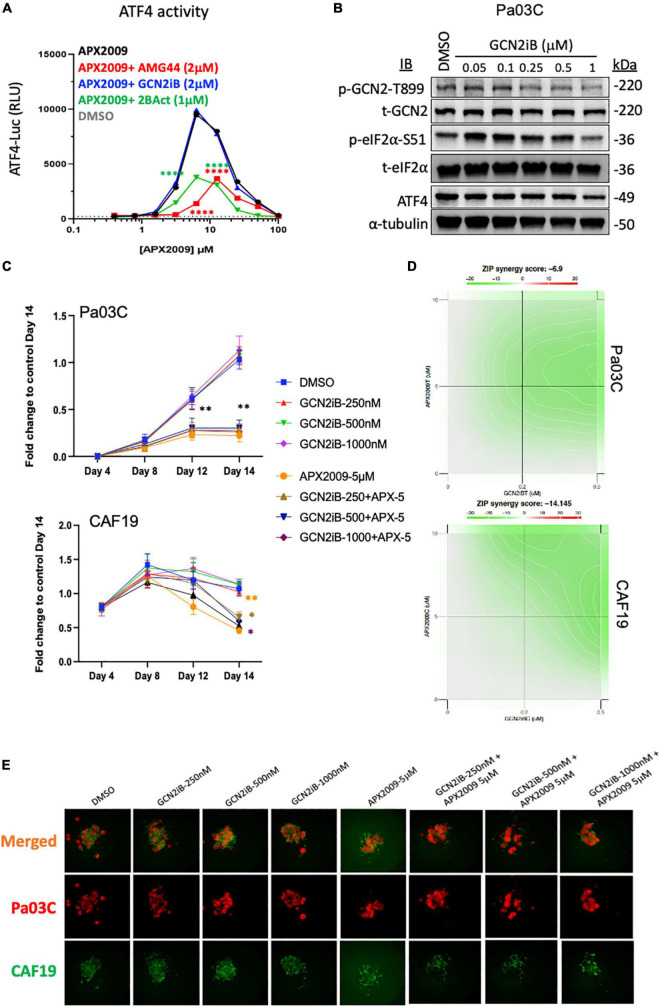
Combination treatment with Ref-1 and general control non-derepressible (GCN) inhibitors results in abrogation of the cell killing effect observed with high doses of protein kinase R-like endoplasmic reticulum kinase (PERK) inhibitor **(A)** HEK293-ATF4-luc reporter cells were treated with APX2009 (4 μM) alone or in combination with AMG-44 (2 μM), GCN2iB (2 μM), or 2BAct (1 μM) for 6 h and luciferase activity was measured. Error bars indicate SE (*N* = 3). Statistical significance was determined using an ordinary two-way analysis of variance (ANOVA) with Tukey’s multiple comparisons test; ****p ≤ 0.0001 compared to APX2009 alone. **(B)** Expression levels of ISR proteins after treatment of Pa03C cells with GCN2 inhibitor, GCN2iB (6 h). α-Tubulin was used as loading control. **(C–E)** The effects of GCN2iB in combination with Ref-1 inhibition were assessed in the 3D co-culture system. Fold change is comparing the total fluorescence intensity at Day 14 of the Media control. Panel **(D)** ZIP synergy calculation showing the effects of inhibitors on tumors as well as on CAF19 determined *via* SynergyFinder 3.0. Synergy scores greater or equal to 10 are synergistic while numbers from –10 to 10 are additive. Panel **(E)** representative images of merged 3D co-culture assays with Pa03C (red) + CAF19 (green) cells following treatment with single agent GCN2iB, APX2009, or the combination. *N* = 3, Student’s *t*-test, *p < 0.05, **p < 0.01 compared to DMSO.

To further test the notion that activation of ISR and GCN2 were critical for the desired response of tumor cell death following Ref-1 inhibition, we utilized a GCN2 inhibitor (GCN2iB) or a GCN2 activator, halofuginone (HF) in combination experiments with Ref-1 inhibition. As shown in Figure 4, activation of p-GCN2 is observed in CAF19, Pa02C, and Pa03C cell lines with high concentrations of AMG-44. To assess the ATF4 transcriptional activity following combination treatment, we treated 293A-ATF4-luc reporter cells with Ref-1 inhibitor APX2009 in combination with inhibitory concentrations of GCN2iB or AMG-44 (2 μM). As expected, Ref-1 inhibition with APX2009 resulted in activation of ATF4 activity. The addition of GCN2iB to the APX2009 treatment did not impact ATF4 activity, while AMG-44 significantly reduced ATF4 transcriptional activity (Figure 6A). ATF4 activity was further increased with high concentrations of AMG-44 alone and in combination with APX2009, again supporting the importance of having the ISR activated for cellular response to Ref-1 inhibition (Figure 4C). There is a decrease in ATF4 luciferase activity when cells are treated with APX2009 in the presence of 2BAct, an allosteric inhibitor of eIF2B (32), indicating that APX2009 induces ATF4 translation resulting in increased ATF4 transcriptional activity (Figure 6A).

Following treatment with increasing concentrations of GCN2iB, there is a dramatic inhibition of p-GCN2 (Figure 6B). Even with potent inhibition of GCN2 with GCN2iB treatment, effects of the single agent therapy on spheroid growth are minimal (Figure 6C). Using concentrations where GCN2 is inhibited in combination with Ref-1 inhibition, the enhancement previously observed with combination therapy are completely abrogated in both cellular compartments (Figures 6C–E). The ZIP synergy model dramatically shifts from +4.9 to −6.9 in tumors and from +14.0 to −14.1 in CAFs (antagonism) (Figure 6D). Blocking activation of GCN2 in combination with Ref-1 inhibition resulted in a reversal of the desired effect of inhibiting 3D spheroid growth (Figure 6E), further supporting that Ref-1 inhibition leads to activation of the ISR which leads to cell death.

**FIGURE 7:**
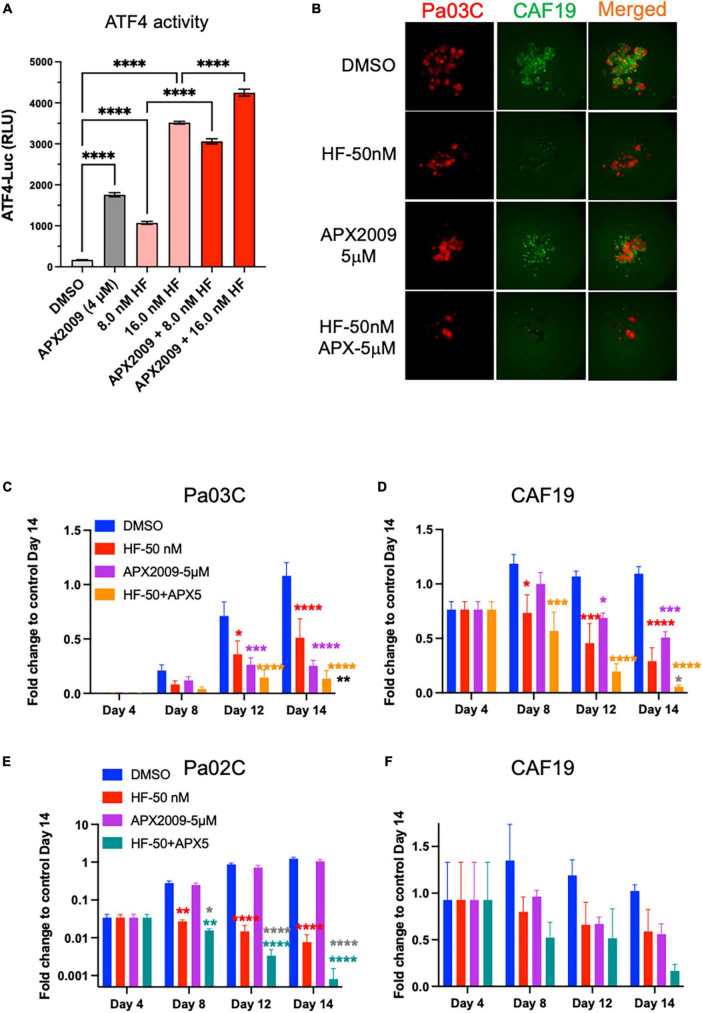
Cell killing following inhibition of Ref-1 redox signaling is enhanced when integrated stress response (ISR) is activated through general control non-derepressible 2(GCN2). **(A)** 293A-ATF4-luc reporter cells were treated with Ref-1 inhibitor APX2009 in combination with halofugione (HF), GCN2 activator for 6 h, and then ATF4 luciferase activity was assessed in the cells. Representative images of 3D co-culture assays with Pa03C (red) + CAF19 (green) cells following treatment with HF (50 nM) in combination with APX2009 (5 μM) are shown in **(B)**. **(C,D)** Spheroid growth of Pa03C + CAF19 was assessed in 3D co-culture system. **(E,F)** Spheroid growth of Pa02C + CAF19 was also assessed in 3D co-culture system. Fold change is comparing the total fluorescence intensity at Day 14 of the Media control to the fluorescence intensity of drug-treated wells. Spheroids were treated with HF in combination with Ref-1 inhibitor, APX2009 5 μM. Error bars indicate SE (*N* = 3–5); Two way analysis of variance (ANOVA) used to generate *p ≤ 0.05, **p ≤ 0.01, ***p ≤ 0.001, ****p ≤ 0.0001, colored symbols is in comparison to DMSO, black symbols are in comparison to HF treatment alone, and dark gray symbols are in comparison to APX2009 treatment alone.

As further proof-of principle that activation of ISR through GCN2 is critical to the observed tumor and CAF effect, we treated the cells with HF, a well-known GCN2 activator, in combination with Ref-1 inhibition (Figure 7, Supplementary Figure 7). Similar to our results with high concentrations of AMG-44, HF also increased ATF4 transcriptional activity following treatment of reporter cells with alone and in combination with APX2009 (Figures 4B, 7A). The cell killing effects of Ref-1 inhibition were again significantly enhanced when cells were co-treated with the GCN2 activator, HF (Figures 7B–F). In Pa03C:CAF19 co-cultures the ZIP synergy values were −11.3 for tumor cells indicating synergy and 7.7 for CAFs indicating additivity. In Pa02C:CAF19 co-cultures, the synergy values were additive in the tumor cells (0.67–1.63) and synergistic in the CAF compartment (1.3–33.7) depending on the dose. Taken together, our data convincingly demonstrated that the effects on tumor killing and TME of Ref-1 inhibition were dependent upon activation of GCN2 and the ISR.

## 4. Discussion

As we seek to understand how the redox function of Ref-1 controls transcription factor DNA binding leading to cellular response to stressors such as nutrient deprivation and hypoxia, tools such as RNA sequencing, single cell RNA-seq, and proteomics were used (6). The relationship between Ref-1 redox signaling and the eIF2 pathway was identified through previous work by our group which demonstrated that Ref-1 knockdown significantly affected the expression of genes associated with the eIF2 signaling pathway using single cell RNA sequencing data (11). However, what is not clear from this study is whether the DNA repair function or the redox signaling function of the Ref-1 protein was responsible for the significant change in gene expression related to the eIF2 signaling pathway. Thus, our study investigated the effect of the redox signaling function of Ref-1 on the activation of the ISR. Ref-1 knockdown findings confirmed our previous findings that the eIF2 signaling pathway is hyperactive under conditions of normoxia and knockdown, but hypoxia alone was enough to activate irrespective of Ref-1 levels (Figures 1C, E).

The effects of Ref-1 signaling and inhibition on the ISR pathway was further delineated here. Previous older reports demonstrated that certain family members of the ATF family (i.e., ATF1 and 2) were under Ref-1 redox control (33). Based on these findings, we surmised that inhibition of Ref-1 redox activity may result in a blockade of ATF family members such as ATF4. To our surprise, the opposite was observed and following treatment with Ref-1 siRNA or selective small molecule inhibitors we observed activation of the ISR pathway as evidenced by eIF2 phosphorylation and activation of ATF4 activity in multiple cell types. Interestingly, the ATF4 activity data in Figure 2F perfectly correlates with the IC_50_ of the APX compounds: APX2014 < APX2009 < APX3330. The negative control analog RN7-58 is a very important control to clearly demonstrate that treating the cells with a naphthoquinone derivative is not non-specifically activating the ISR, but that this response is due to a blockade in Ref-1 redox activity. We have previously demonstrated that APX2009, APX2014, and APX3330 can generate ROS, but RN7-58 does not (9). It is noteworthy that PERK has been suggested to contribute to NRF2 activation by direct phosphorylation during ER stress (34). Moreover, Fishel et al. have demonstrated that blockade of Ref-1 redox signaling activates NRF2 and its downstream targets, such as HMOX1 in multiple human PDAC lines (35). Our results suggest that PERK may contribute to the oxidative stress response induced by treatment with Ref-1 inhibitors. Future work will include the addition of ROS scavengers to see if this can block the activation of the ISR in combination with Ref-1 inhibitors.

Furthermore, knockdown of each of the four ISR kinases implicated PERK as the main ISR kinase responsible for the activation of the pathway following inhibition of Ref-1 redox signaling (Figure 2). However, knockdown of PKR and HRI also affected the levels of p-eIF2α. Our and others’ previous data has shown that Ref-1 inhibition results in mitochondrial dysfunction and HRI has also been implicated in sensing of mitochondrial stress indicating another possibility of crosstalk between Ref-1 redox signaling and activation of ISR (6, 7, 36). These data do demonstrate a strong and reproducible activation of the ISR pathway and that it is playing a major role in the tumor cells’ response to Ref-1 inhibition. Due to these findings, the activation of the ISR could be evaluated *in vivo* as well as in patient samples prior to and following treatment with Ref-1 inhibitors. Perhaps these findings could lead to correlation of ISR pathway activation as a biomarker of response to Ref-1 blockade and response to treatment. The phosphorylation of eIF2α results in gene programming which allows the cell to recover from stress-induced damage facilitating survival as well as promoting apoptosis in response to chronic stress (37). Numerous studies have revealed that the PERK-eIF2α pathway is activated under hypoxic conditions in both *in vivo* and *in vitro* studies, and that this pathway is also essential for optimal tumor growth (38). Therefore, we hypothesized that the activation of ISR following Ref-1 inhibition could be leading to two scenarios: promoting resistance to these inhibitors or the mechanism by which cell death is induced.

With several well-characterized inhibitors of the ISR pathway available and PERK phosphorylation of eIF2 important following APX2009 treatment, we wanted to investigate whether blocking the activity of PERK would lead to an increase in the cells’ response to Ref-1 inhibitors. Increasing doses of PERK inhibitor, AMG-44 demonstrated a biphasic response in terms of the activation of eIF2α. At low doses in which PERK was inhibited, eIF2 phosphorylation and ATF4 activity were also inhibited. However, at high concentrations of AMG-44, we observed activation of ATF4 activity and increased phosphorylation of eIF2α through GCN2. AMG-44 is an ATP competitive inhibitor of PERK with a type I 1/2 binding mode (16). We and others have shown that type I 1/2 and type II inhibitors activate GCN2 at critical concentrations (39–41). It is suggested that these inhibitors bind to one protomer in the GCN2 kinase domain dimer and stimulate the kinase activity of the adjacent protomer (41). As reported here, AMG-44 appears to have this property as well. The combination data in 3D co-culture spheroids in which we can monitor both tumor and CAF intensity implied that ISR needed to be active for full cell killing effect following Ref-1 inhibition. To explore this further as well as further understand the role of GCN2, we used GCN2 activator, HF and GCN2 inhibitor, GCN2iB in combination with Ref-1 inhibitors in our 3D co-culture assay. These combination experiments further demonstrated that both tumor cells and CAFs have a greater response to APX compounds when the ISR is active: HF + APX2009 resulted in enhanced cell killing and GCN2iB + APX2009 abrogated the effects of killing in both tumor and CAFs. Taken together, our findings indicate that combination therapy with high doses of PERK inhibitor AMG-44 results in p-GCN2 activation which in turn induces the ISR to drive apoptosis in response to chronic cell stress through inhibition of Ref-1.

Another important aspect of this study that is worth discussing is the use of these relevant *in vitro* models consisting of tumor and stroma to study tumor adaptation to stress and response to therapeutics. Interestingly, the effects of PERK inhibition or GCN2 inhibition were minimal on both tumor and CAF lines in monolayer, however, inhibition of cell growth was observed in the 3D co-culture assay. The CAF19 cells appear to be more sensitive to inhibition of PERK and GCN2 in comparison to tumor cells and this was more apparent in the 3D co-culture assay. CAFs are an important contributor to the tumor’s response to stress as well as treatment, and the CAF population is also heterogeneous leading to differential effects on the extracellular matrix, recruitment of other cell types within the tumor microenvironment, and stiffness (42–44). ATF4 has been implicated in driving CAF activation affecting pathways of collagen synthesis as well as angiogenesis (45). Furthermore, HF has been used *in vivo* as a stromal reprograming agent in PDAC studies (46, 47). This data alongside our data in Figure 7 supports the use of HF or other similar GCN2 activating agents and Ref-1 inhibitors as a therapeutic regimen in preclinical pancreatic cancer models. It is important to note that the effects that we observe in these studies with the APX compounds and AMG-44 are in concentration ranges that are readily achievable in pre-clinical models (16). Our group has shown that we can get significant μM amounts of parent drug APX3330 in patient serum (20–120 μM) as observed in our clinical trials (NCT03375086 and NCT04692688).

This is the first study to identify that Ref-1’s redox signaling activity plays a crucial function in the cellular stress response by specifically activating the ISR. Through the integration of ATF4 activity assays, phospho-specific antibodies to indicate activity, 3D co-culture assays, and the use of selective small molecule inhibitors of Ref-1, PERK, and GCN2, we have discovered that the co-culture of pancreatic cancer cells and CAFs respond to the blockade of Ref-1 redox signaling through phosphorylation of PERK and eIF2α. This activation of the ISR leads to the desired response of tumor and stromal cell growth reduction. Therefore, it would be of interest to carry out additional preclinical studies to investigate combination regimens that consist of Ref-1 inhibitors with compounds that activate the ISR. Several kinase inhibitors, including the FDA-approved agent Neratinib, have been shown to activate GCN2 (39–41). Moreover, peak plasma levels of Neratinib would be sufficiently high enough to induce GCN2 in patients (41). In yet another interesting example of activation of the ISR, in pancreatic cancer, the proteasome inhibitor Bortezomib was shown to activate HRI which was suggested to enhance sensitivity to this proteasome inhibitor (48). Future studies will focus on evaluating combinations with inhibitors of Ref-1 and clinically relevant GCN2 activators or other novel ISR activators in *in vivo* models, which will provide additional therapeutic options for this deadly disease.

## Data availability statement

The original contributions presented in this study are included in the article/Supplementary material, further inquiries can be directed to the corresponding authors.

## Author contributions

MM performed the experiments, data validation, formal analysis, and writing and editing the manuscript. MB and SG performed the experiments and assays and writing. EK, RC, and AJK performed the experiments and assays. CZ performed the bioinformatic analysis, formal analysis, and editing. KS performed the experiments, data analysis, and writing and editing the manuscript. MK provided expertise, access to APX drugs, experimental design, and writing/editing. KS and MF provided expertise, performed the experiments, experimental design, analysis, funding, and writing/editing of manuscript. All authors contributed to the article and approved the submitted version.

## Footnotes

1
https://synergyfinder.fimm.fi/synergy/synfin_docs/#reference
