# Supplementary material for: Activation of the integrated stress response (ISR) pathways in response to Ref-1 inhibition in human pancreatic cancer and its tumor microenvironment
Source: Front Med (Lausanne). 2023 Apr 27;10:1146115. doi: 10.3389/fmed.2023.1146115 (PMC10174294; doi:10.3389/fmed.2023.1146115)
Supplement: Supplementary file 1 [file Data_Sheet_1.docx]

Mock

SCR

siRef-1

Mock

SCR

siRef-1

Normoxia

Hypoxia

p-PERK-T982

150 kDa

Vinculin

117 kDa

p-GCN2-T899

240 kDa

t-PERK

150 kDa

240 kDa

t-GCN2


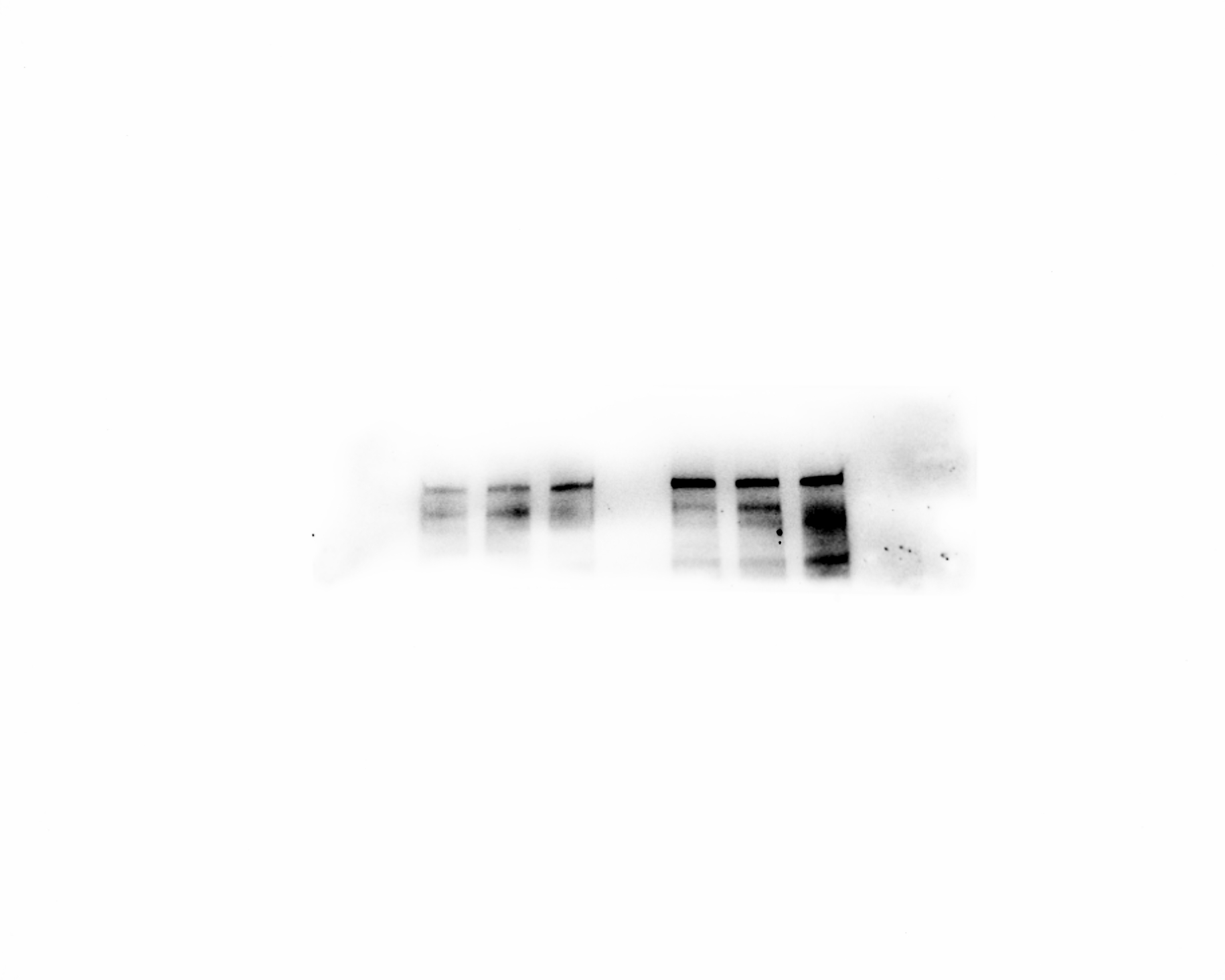

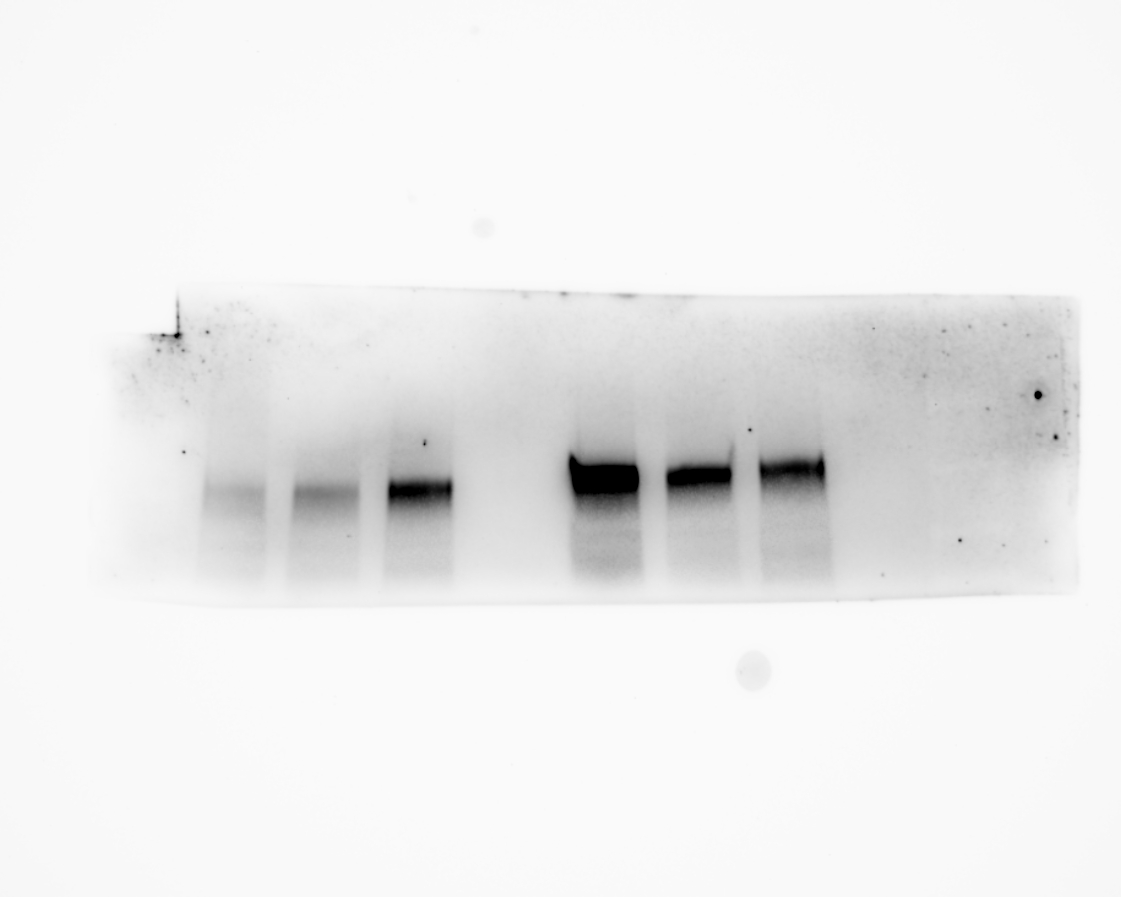

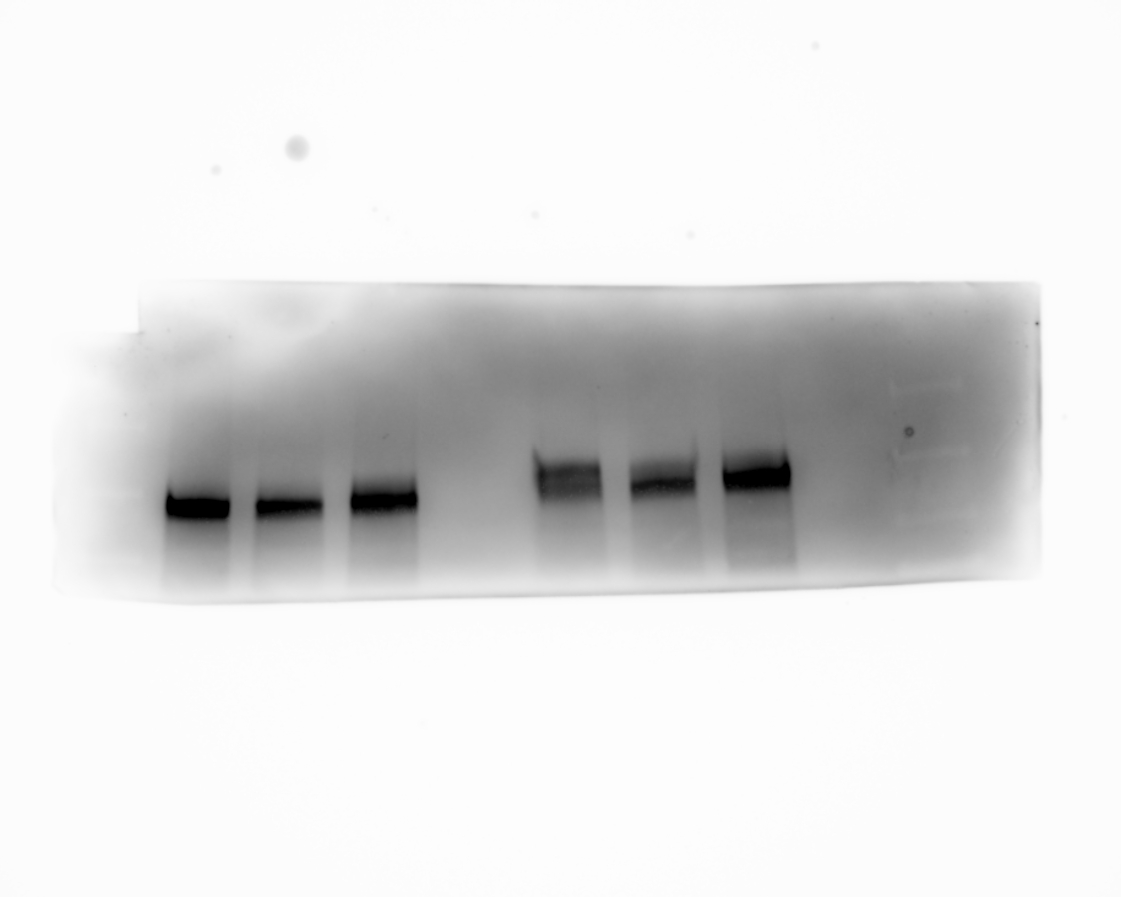

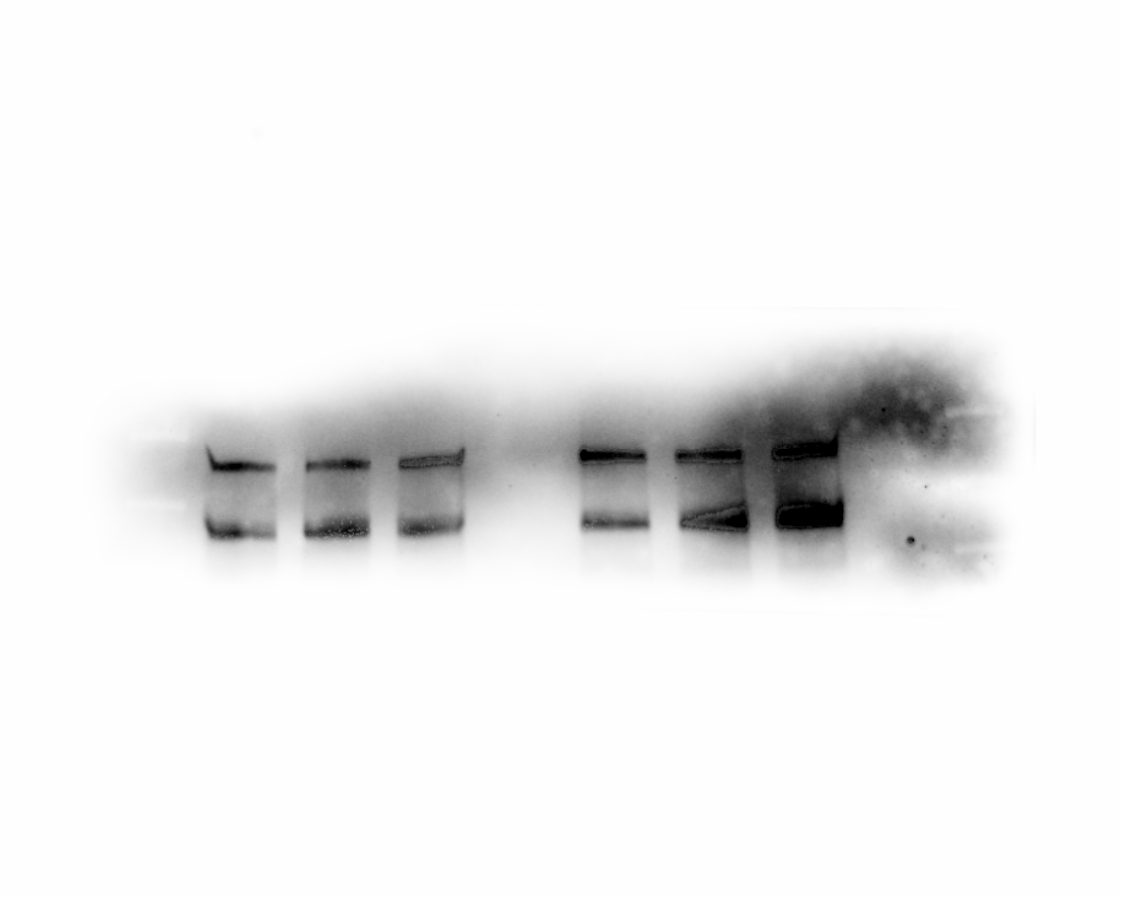

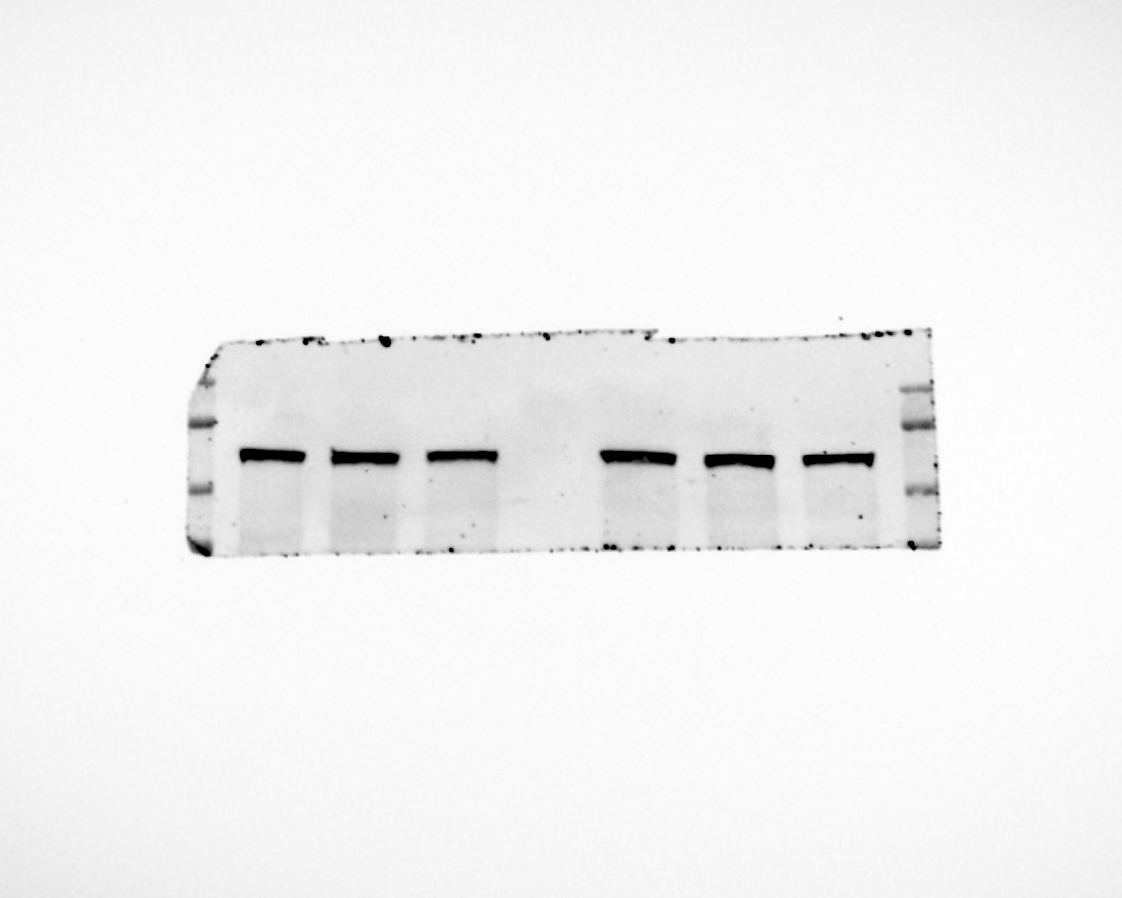


**Supplemental Figure S1.** Expression levels of ISR biomarkers after Ref-1 KD in human PDAC cells under normoxia and hypoxia (1% O_2_, 24h), Vinculin was used as loading control and is the same loading control as in Figure 1C for consistency, N=3.


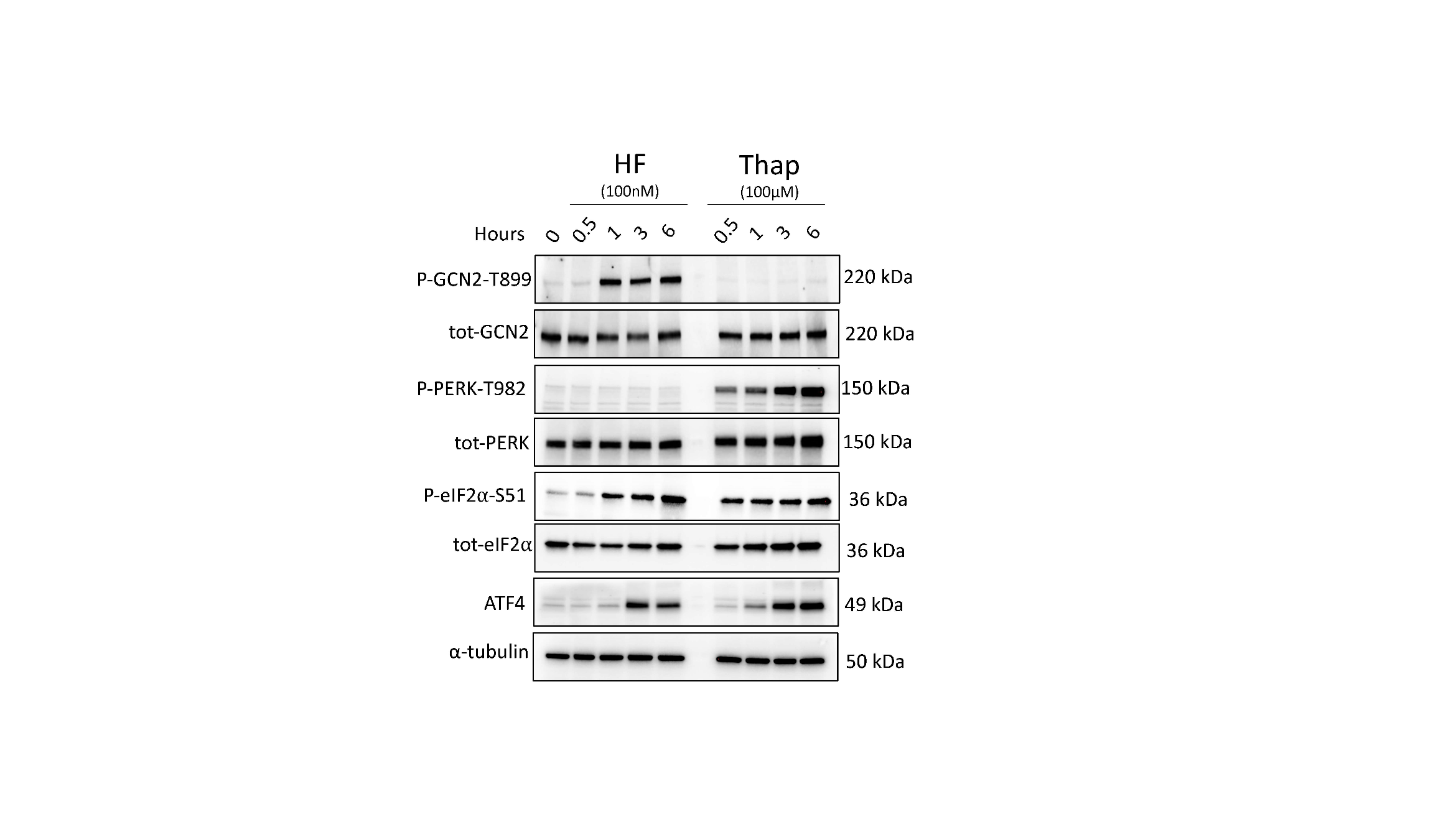


**Supplemental Figure S2. Treatment with Ref-1 inhibitors as well as known ISR activators demonstrate robust activation of ISR.**

Pa03C cells were treated with HF (Halofuginone) and Thap (Thapsigargin) and the expression levels of ISR biomarkers in different time points were assessed, two independent experiments were performed.


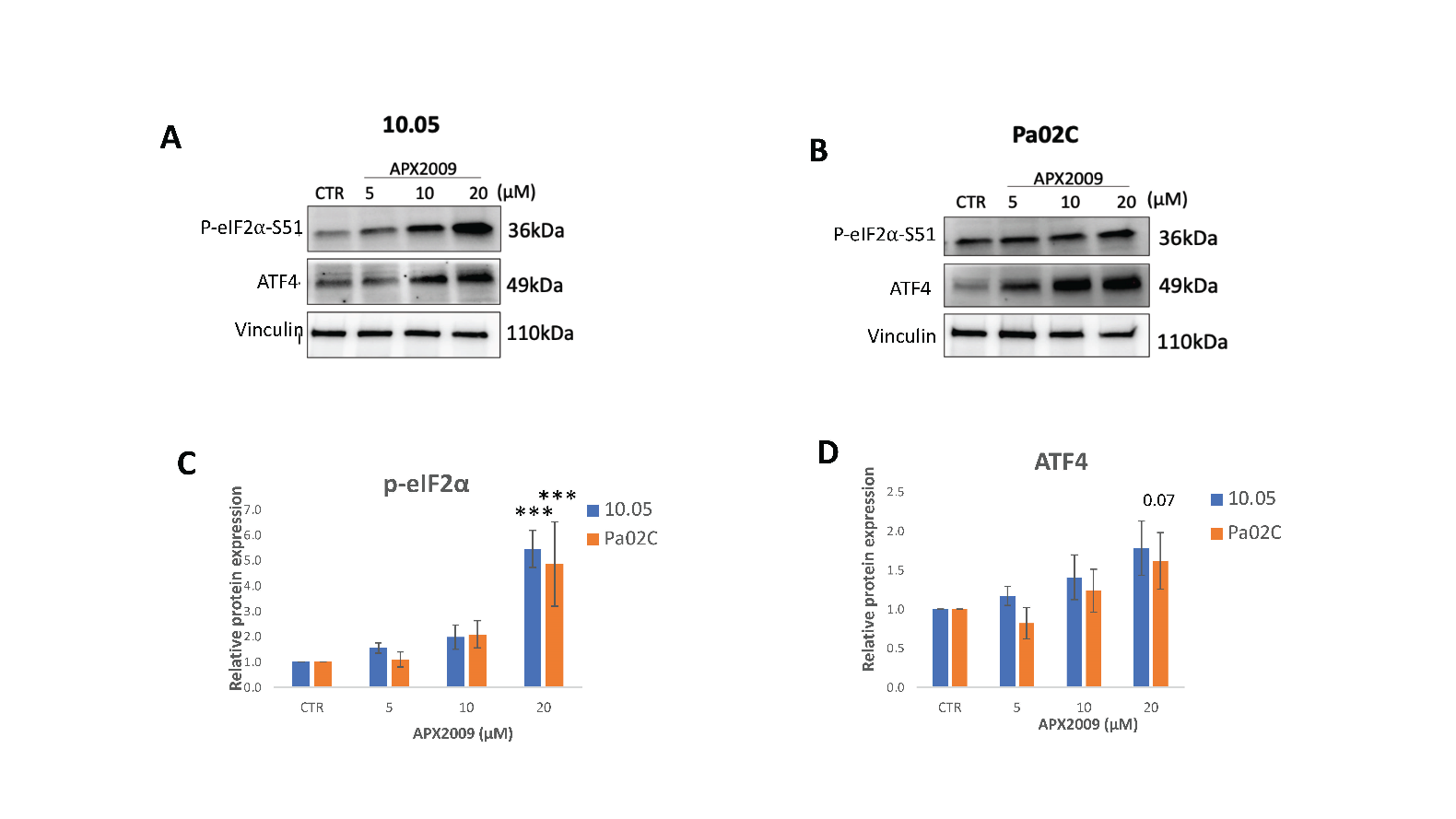


**Supplemental Figure S3. Effects of inhibition of Ref-1 redox signaling in activation of integrated stress response in additional human PDAC cell lines.** A-B) Additional human PDAC cell lines (10.05 and Pa02C, respectively) treated with different concentrations of APX2009 for 6h. Vinculin was used as loading control. C-D) Quantification of p-eIF2⍺ and ATF4 were shown in Figure 4C-D. One-way ANOVA was used for statistical analysis:^*^ ^***^p<0.001. Three independent experiments were performed (N=3).

**
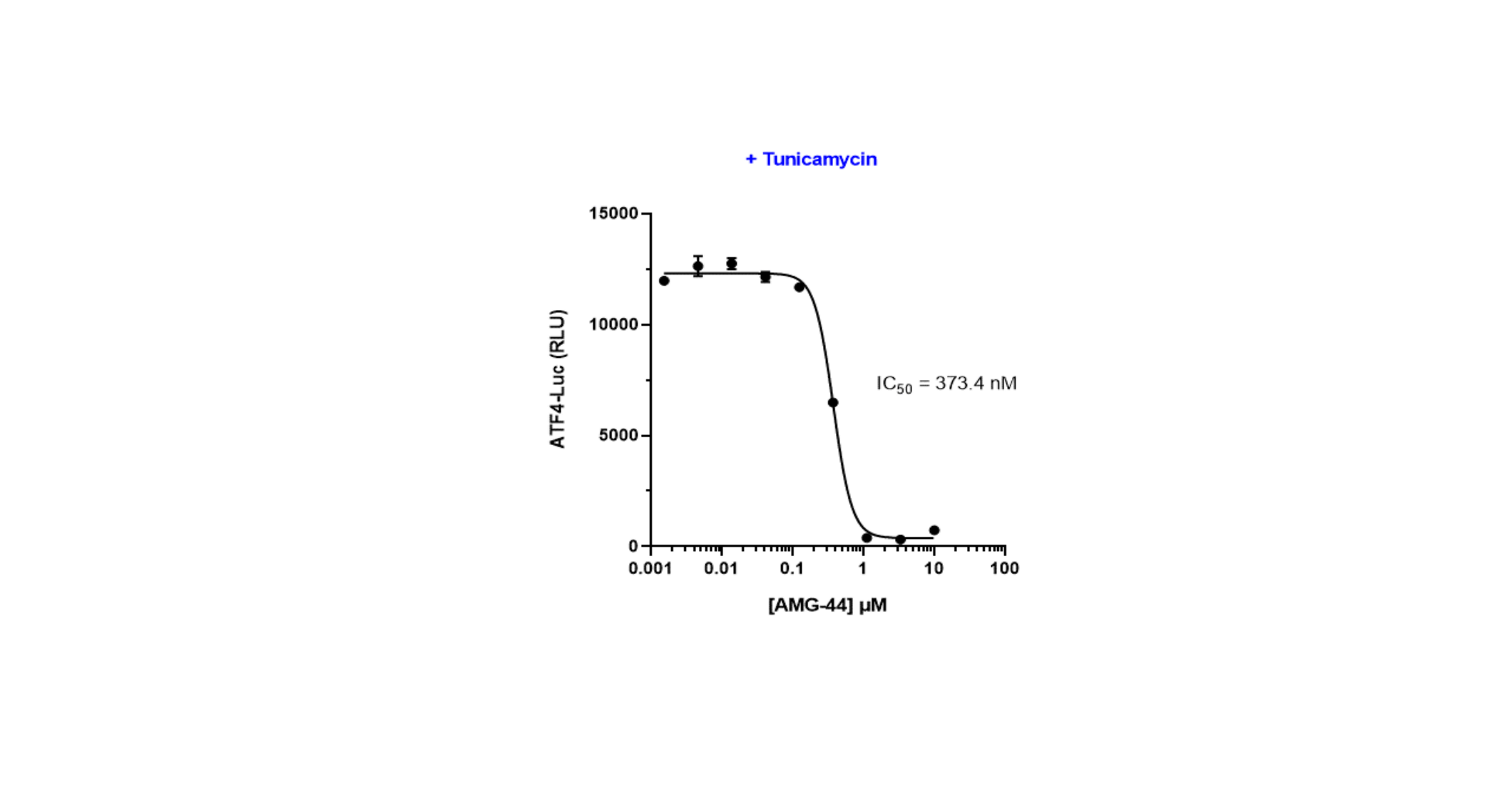
**

**Supplementary Figure S4. AMG-44 inhibits PERK and the ISR.** HEK293A-ATF4-luc reporter cells were treated with AMG-44 as indicated in the presence of 2 µM tunicamycin to induce ER stress and luciferase activity was measured after 6 hours. The calculated IC50 from the fitted inhibition curve is indicated.


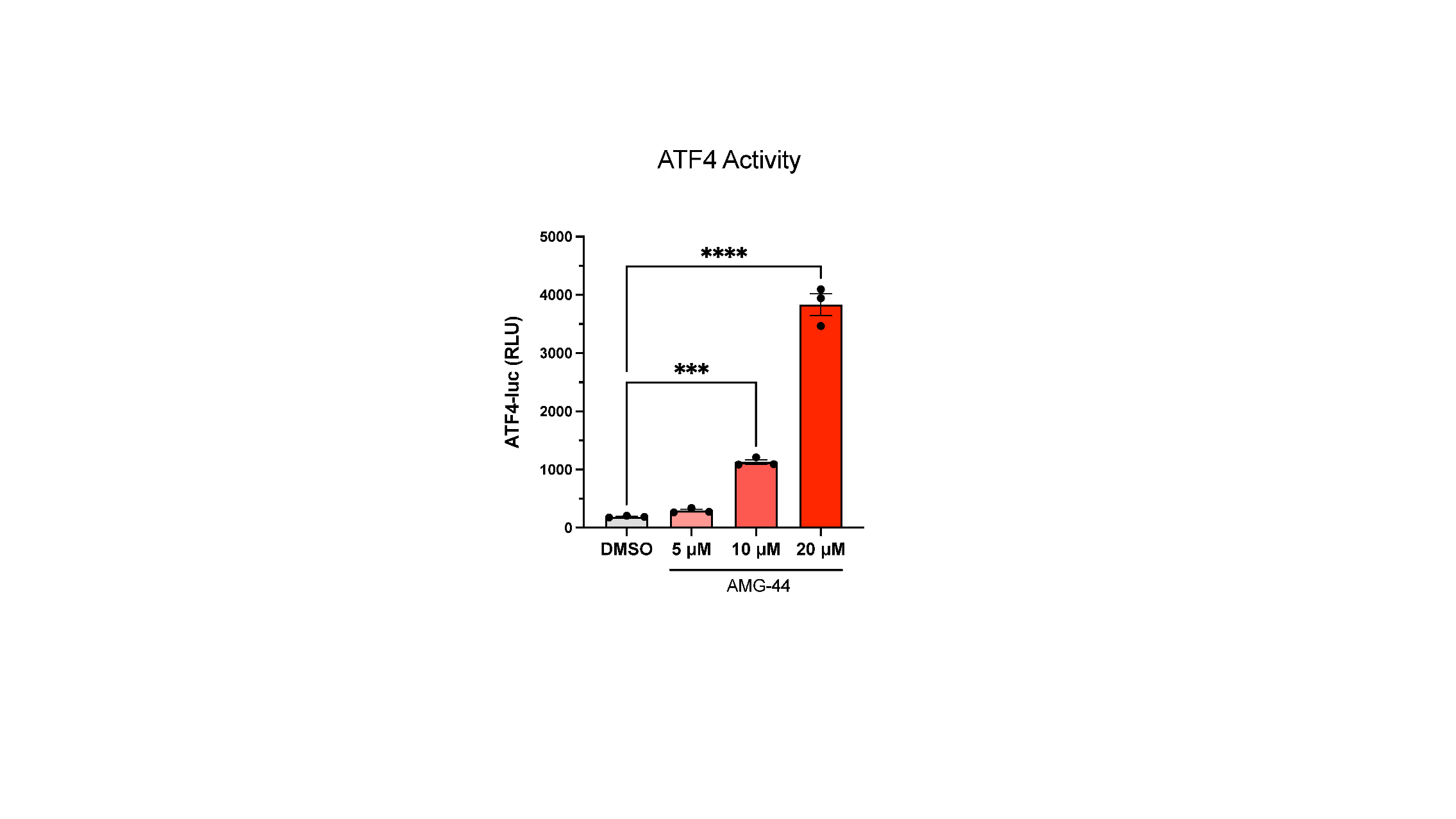


**Supplementary Figure S5. Effects of AMG-44 on ATF4 activity.**

HEK293A-ATF4-luc reporter cells were treated with AMG-44 as indicated concentrations, and luciferase activity was measured after 6 hours. Student T-test was used, ***p<0.001, ****p<0.0001.


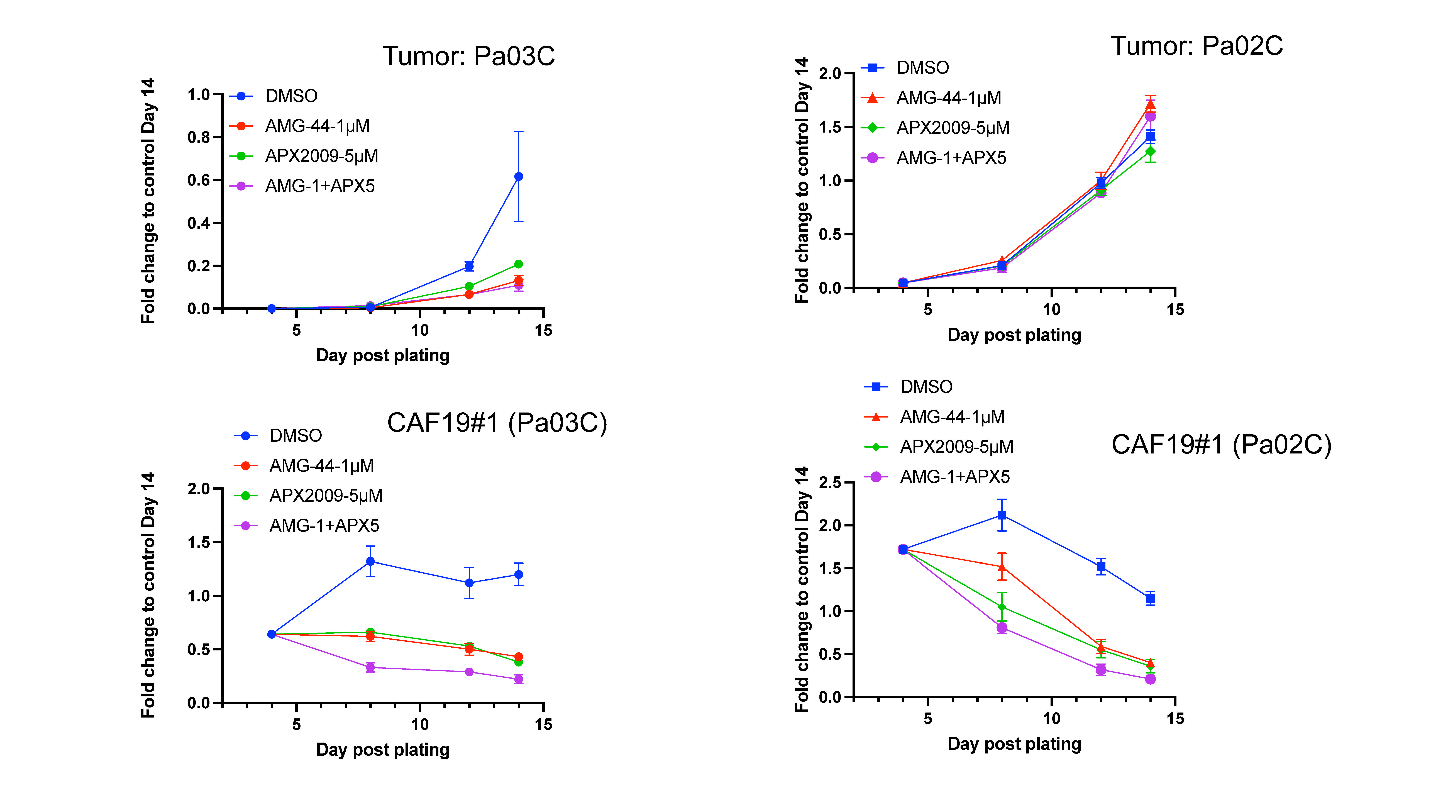


**Supplementary Figure S6.** **At doses of AMG-44 that are inhibitory to PERK in combination with Ref-1 inhibition, there is no enhancement of spheroid growth.**

Co-cultures of Pa03C+CAF19 (left) or Pa02C+CAF19 (right) are plated, scanned, and treated on Days 4, 8, and 11. Final scan is on Day 14. Quantitation of the fluorescent intensity of tumor cells expressing TdTomato, and CAFs expressing EGFP is shown over time. Normalization of total intensity is done by comparing treated well intensity to Media control wells on Day 14. 3D co-culture assays with Pa03C or Pa02C + CAF19 cells following treatment with AMG-44 (1µM, red) or APX2009 (5 μM, green) alone or in combination (purple) is shown over time. Vehicle control is DMSO (blue).


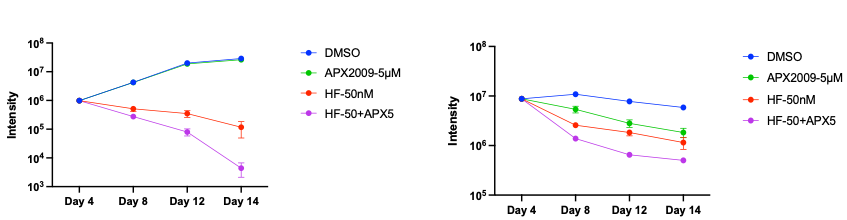


**Supplementary Figure S7. Combination of Ref-1 inhibition and stimulation of ISR through GCN2 is more effective at killing tumor and CAF cells.**

Co-cultures of Pa02C+CAF19 are plated, scanned, and treated as previously described. Quantitation of the fluorescent intensity of tumor cells expressing TdTomato, and CAFs expressing EGFP is shown over time. 3D co-culture assays with Pa02C + CAF19 cells following treatment with HF (50nM, red) or APX2009 (5 μM, green) alone or in combination (purple) is shown over time. Vehicle control is DMSO (blue).

| Table 1S. Chemicals and reagents that are used for experiments. | | |
| --- | --- | --- |
| **Chemicals and Reagents** | **Sources** | **Catalog Number** |
| APX2009 | Apexian Pharmaceuticals | --- |
| APX2014 | Apexian Pharmaceuticals | --- |
| RN7-58 | Apexian Pharmaceuticals | --- |
| Halofuginone (HF) | Caymen | Cat #13370 |
| Thapsigargin (Thap) | Sigma | Cat #T9033 |
| GSK2656157 | MedChemExpress | HY-13820 |
| GCN2iB | MedChemExpress | HY-112654 |
| AMG-44 | MedChemExpress | HY-12661A |
| 2BAct | MedChemExpress | HY-125021 |
|  | | |
|  |  |  |

| Table 2S. Antibodies that are used for the experiments. | | |
| --- | --- | --- |
| **Antibodies** | **Sources** | **Catalog Number** |
| p-GCN2 | Abcam | Cat #ab75836 |
| Total GCN2 | Cell Signaling | Cat #3302S |
| p-PERK | Custom | PMID: 26130148 |
| Total PERK | Cell Signaling | 3192S |
| p-eIF2α | Abcam | ab323157 |
| Total eIF2α | Cell Signaling | Cat #5324S |
| ATF4 | Cell Signaling | Cat #11815S |
| PKR | Cell Signaling | CS-12297S |
| Ref-1 | Novus | 13B8E5C2 |
| Vinculin | Millipore | CP74-100 |
| α-tubulin | Abcam | ab7291 |
|  |  |  |

| **Table 3S. siRNAs that are used for the knock down (KD) experiments.** | | |  |
| --- | --- | --- | --- |
| **siRNAs** | **Sequence** | **Vendor** | **Catalog number** |
| SCR_Ref-1 | 5′-CCAUGA GGUCAGCAUGGUCUG-3′, 5′-GACCAUGCUGACCUCAUGGAA-3′ | Dharmacon | CTM-253996 |
| Ref-1 | 5′-GUCUGGUACGACUGGAGUACC-3′, 5′UACUCCAGUCGUACCAGACCU-3′ | Dharmacon | [6, 7, 21, 35] |
| SCR_Universal | UGGUUUACAUGUCGACUAA | Dharmacon | D-001810-10-20 |
| PERK-3 | GCAAUUAGCCUUAAGUUGU | Dharmacon | J-004883-11 |
| GCN2-2 | GCAAUUCUGUGGUGCAUAA | Dharmacon | J-005314-06 |
| PKR-2 | GCGAGAAACUAGACAAAGU | Dharmacon | J-003527-10 |
| HRI | GCAGAAAUCCAGGUGUUAA | Dharmacon | J-005007-07 |
|  |  |  |  |

| **Table 4S. qPCR primers.** | | |
| --- | --- | --- |
| **Primers** | **Forward** | **Reverse** |
| HRI | CTCAGGCGTAATTCCCACCTAG | TGGATGTGCAGCATCAGGTGGT |
| β-actin | CACCATTGGCAATGAGCGGTTC | AGGTCTTTGCGGATGTCCACGT |
|  |  |  |
